# Neutrophil Elastase-mediated proteolysis activates the anti-inflammatory cytokine IL-36 Receptor antagonist

**DOI:** 10.1038/srep24880

**Published:** 2016-04-22

**Authors:** Tom Macleod, Rosella Doble, Dennis McGonagle, Christopher W. Wasson, Adewonuola Alase, Martin Stacey, Miriam Wittmann

**Affiliations:** 1 School of Molecular and Cellular Biology, Faculty of Biological Sciences, University of Leeds, Leeds, UK; 2 Leeds Institute of Rheumatic and Musculoskeletal Medicine (LIRMM), University of Leeds, UK; 3 National Institute of Health Research (NIHR) LMBRU, Chapel Allerton Hospital, Leeds; 4 Centre for Skin Sciences, Faculty of Life Sciences, University of Bradford, UK

## Abstract

The interleukin-36 receptor antagonist (IL-36Ra) which regulates IL-36α, -β and -γ is linked to psoriatic inflammation, especially loss-of-function mutations in pustular psoriasis subtypes. As observed with other IL-1 superfamily proteins, the IL-36 members require N-terminal cleavage for full biological activity but the mechanisms of IL-36Ra activation remain poorly defined. Using different blood leukocyte and skin resident cell preparations, and recombinant proteins, we have identified that neutrophil elastase, but not other neutrophil derived proteases, cleaves IL-36Ra into its highly active antagonistic form. The activity of this processed form of IL-36Ra was confirmed in human primary dermal fibroblasts and keratinocytes and in skin equivalents. A significant dose dependent reduction of IL-36γ induced IL-8 and chemokine ligand 20 (CCL20) levels were detected following addition of the cleaved IL-36Ra compared to full length IL-36Ra. By activating IL-36Ra, the neutrophil derived protease can inhibit IL-36 induced chemokine production, including IL-8 and CCL20, and reduce further inflammatory cell infiltration. These findings strongly indicate neutrophil elastase to be a key enzyme in the biological function of IL-36Ra and that neutrophils can play a regulatory role in psoriatic inflammation with regard to balancing the pro-inflammatory activity of IL-36.

Psoriasis is an immune-mediated inflammatory disease affecting approximately 2% of the Caucasian population^1^. Psoriatic inflammation ranges broadly in both form and severity, presenting as localised plaques, arthritis, pustular lesions or systemic inflammatory outbreaks^2^. Psoriasis is characterised by hyperkeratotic plaques with increased capillary networks in the upper dermis, accompanied by epidermal inflammatory cell infiltrates, culminating in lesions with a raised red and scaly appearance.

Despite the documented role that T cells and the adaptive immune system play in psoriasis, recent research has established a crucial role for the innate inflammatory mediators, including IL-1 family members, tumour necrosis factor α (TNFα), IL-12, IL-23, IL-17C and IL-20 family members in initiating and maintaining psoriatic inflammation^3^,^4^. Examination of mRNA and protein has revealed the IL-36 cytokines to be amongst the most highly up-regulated and disease specific genes expressed in psoriatic plaques^5^. The IL-36 cytokines are members of the IL-1 super-family, consisting of the agonists IL-36α, IL-36β and IL-36γ, and an antagonist IL-36Ra. Analogous to other members of the IL-1 family, the agonists bind a specific receptor (IL-36R), causing recruitment of IL-1 receptor accessory protein (IL-1RAcP) to facilitate signal transduction via myeloid differentiation 88 (MyD88) and TNF receptor-associated factor 6 (TRAF6), ultimately resulting in activation of the nuclear factor NF-κB and expression of a host of pro-inflammatory mediators^6^. The receptor antagonist (IL-36Ra) binds the same IL-36R but does not lead to accessory protein recruitment and the consequent signalling, thereby competing with and inhibiting the activity of the other IL-36 cytokines^7^. Overexpression of IL-36α in keratinocytes of K14-IL-36α transgenic mice leads to the development of transient inflammatory skin symptoms in mice, and leaves them highly susceptible to development of psoriatic-like plaques upon treatment with 12-O-tetradecanoylphorol-13-acetate. Indeed, eluding towards the potential importance of the IL-36 family in psoriasis, these mice act identically on a Rag2^−/−^ background where there are no T cells to affect the development of the plaques. Using the same model, IL-36Ra deficient mice exhibited chronic skin abnormalities and exacerbated plaque development, demonstrating the importance of IL-36Ra in the regulation of IL-36 mediated inflammation^8^. Of pivotal importance is the observation that mutations of *IL36-RN* (gene encoding IL-36Ra) have also been shown to cause generalised pustular psoriasis (GPP), the most severe and potentially life threatening form of psoriatic inflammation^9^.

Like IL-1 and other members of the IL-1 family the activity of IL-36 cytokines is strictly regulated through the N-terminal cleavage of the nascent peptides. Although the active forms of the cytokines have not yet been isolated *in vivo*, it has been demonstrated that precise cleavage 9 amino acids upstream of a common A-X-D motif is crucial for activation. In the case of IL-36Ra, this is achieved by removal of the N-terminal methionine, generating the active IL-36Ra V2 form of the protein possessing an N-terminal valine residue^7^. Recent crystallographic data reveal that without this stringent cleavage, it’s likely that N-terminal overhang interferes with receptor binding^10^. However, unlike others of the IL-1 family, the IL-36 proteins contain no caspase-1 cleavage motif, and the amino acid sequence surrounding the cleavage sites bear little homology to one another.

As a result, despite understanding where cleavage must occur, the responsible physiological proteases have remained elusive. It has been postulated before that as the sequences surrounding the cleavage sites are markedly different, a number of different proteases may be responsible for their cleavage. The activity of these proteases may therefore be critical in regulating the physiological and pathological roles of the IL-36 cytokines. Through the use of proteases derived from cells present at psoriatic lesions we show that neutrophil elastase is capable of cleaving IL-36Ra into its mature anti-inflammatory form. Given that a balance must exist between elastase and its inhibitors, it seems logical that disturbances in such a balance might influence the ratio of active to inactive IL-36Ra and hence may be exploited to effect the regulation of IL-36 mediated inflammation.

## Results

### Polymorphonuclear cell supernatant cleaves IL-36Ra

**Figure 1:**
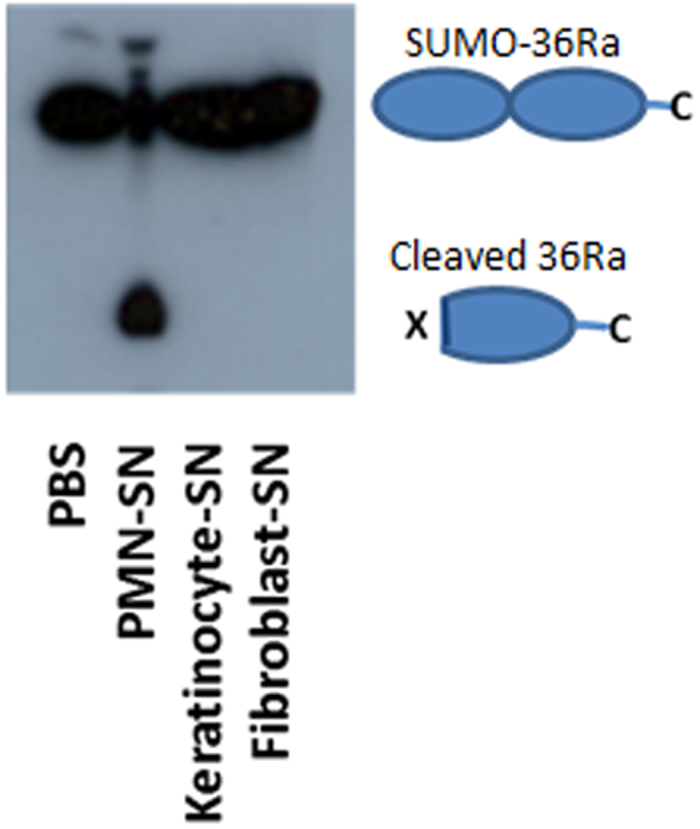
SUMO tagged IL-36Ra cleavage by activated cellular supernatants. SUMO tagged IL-36Ra was incubated with a range of supernatants from activated cells. PMNs were isolated from whole blood of healthy donors and then stimulated with 100 ng/ml of PMA for 1 hour at 37 °C. Human primary keratinocytes and fibroblasts were stimulated with 100 ng/ml of PMA for 24 hours at 37 °C. Supernatants were removed from the cells and incubated with IL-36Ra for 1 hour at 37 °C, along with a PBS control. Samples were subjected to WB analysis.

IL-36 cytokines are expressed in the skin, lungs and joints, and since the skin is the primary site of psoriasis it seemed logical that IL-36 proteins may be cleaved to their active forms in the skin compartment, and that therefore a protease cleaving the proteins to full activity would also be expressed here. Small Ubiquitin-like Modifier (SUMO) tagged IL-36Ra was therefore incubated with supernatants from fibroblasts, keratinocytes and polymorphonuclear cells (PMNs); all cells expected to be found in lesional psoriatic skin. Western blot (WB) analysis showed supernatant from PMNs stimulated with phorbol myristate acetate (PMA) cleaved IL-36Ra, whilst in this experimental setup no cleavage was evident from incubations with fibroblast or keratinocyte derived supernatants (Fig. 1).

### A serine protease is responsible for the cleavage of IL-36Ra

**Figure 2:**
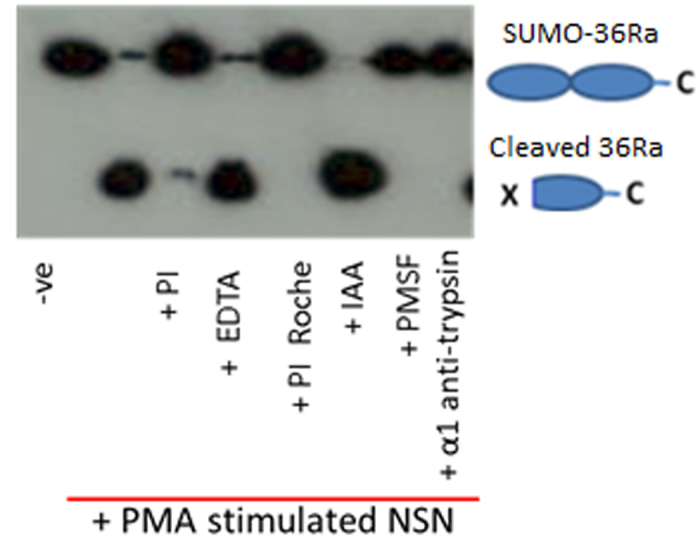
IL-36Ra cleavage by activated PMN supernatant is prevented by serine protease inhibitors. SUMO tagged IL-36Ra was incubated with supernatant from PMNs stimulated with PMA for 1 hour at 37 °C. Incubation was performed in the presence or absence of a range of protease inhibitors. PI = complete protease inhibitor cocktail (Roche), EDTA = Ethylenediaminetetraacetic acid, inhibitor of proteases where a metal ion is required for cleavage, PI Roche = complete ultra-protease inhibitor cocktail which includes aspartic proteases (Roche), IAA = iodoacetic acid a pan cysteine protease inhibitor, PMSF = phenylmethylsulfonyl fluoride a pan serine protease inhibitor, α1 anti-trypsin is another pan serine protease inhibitor. Samples were then analysed using WB.

As PMN supernatant was shown to cleave IL-36Ra, further incubations with stimulated PMN supernatant were performed to reduce the number of candidate proteases. Incubating in the presence or absence of pan-protease inhibitors (1x PI Roche), phenylmethanesulfonylfluoride (PMSF) (1 mM), iodoacetic Acid (IAA) (1 μM), ethylenediaminetetraacetic acid (EDTA) (500 μM) and α1 anti-trypsin (1 mg/ml) was used to identify which protease family the active protease belongs to. WB analysis of incubations revealed that in the presence of IAA and EDTA, inhibitors of cysteine and metalloproteinase proteases, cleavage occurred. However, incubation with PMSF and α1 anti-trypsin, prevented processing indicating that the cleavage was due to a serine protease (Fig. 2).

### Neutrophil elastase cleaves IL-36Ra to its mature IL-36Ra V2 form

**Figure 3:**
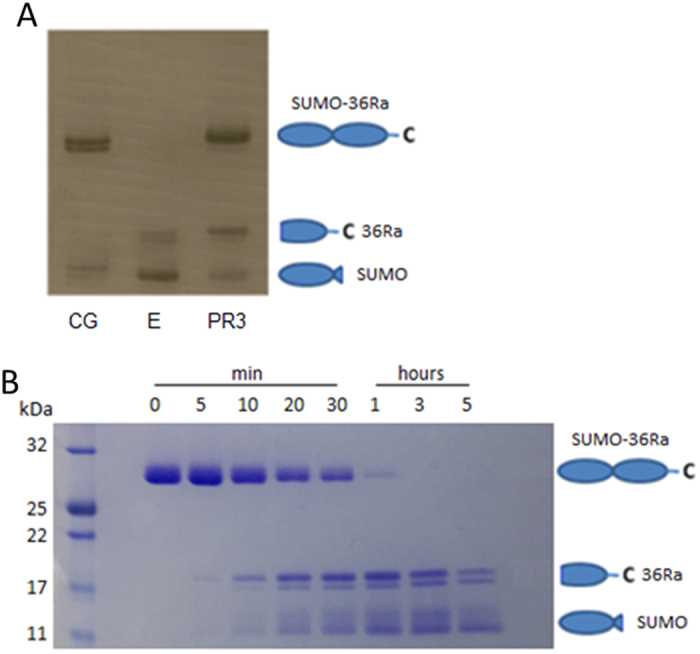
IL-36Ra is efficiently cleaved using recombinant neutrophil serine proteases. (**A**) SUMO tagged IL-36Ra was incubated with the three most abundant neutrophil serine proteases, cathepsin G (CG), elastase (E) and proteinase 3 (PR3) at 1/100 of IL-36Ra added for 10 minutes at 37 °C. Coomassie stained gel shows samples following incubation. (**B**) SUMO tagged IL-36Ra was incubated with elastase at 1/100 of IL-36Ra added over a period of 5 hours at 37 °C. Coomassie stained gel shows samples taken at various time points during incubation.

Since a PMN serine protease was identified to cleave IL-36Ra, the SUMO tagged protein was incubated with a number of recombinant serine proteases expressed by neutrophils. Incubations were performed with cathepsin G, neutrophil elastase, and proteinase 3 at a ratio of 1:100 protease to protein. Analysis of the incubations by SDS-PAGE indicated that neutrophil elastase efficiently cleaved IL-36Ra, compared to proteinase 3 and cathepsin G which had much lower cleavage activity (Fig. 3). Further analysis of the cleaved products by mass spectrometry and N-terminal sequencing identified the truncations generated (shown in table 1) and revealed that elastase cleaved IL-36Ra precisely 9 amino acids upstream of its A-X-D motif to the mature IL-36Ra V2 form, whilst prolonged incubation with cathepsin G and proteinase 3 generated a cleavage product with an N-terminal serine (IL-36Ra S4). In order to show these truncations were not the product of fusion protein artefacts, identical incubations using full length proteins lacking N-terminal SUMO tags were performed. These generated the same cleavage products (results not shown).

### IL-36Ra V2 regulates IL-36 mediated inflammation in primary fibroblasts and keratinocytes and an epidermal skin model

**Figure 4:**
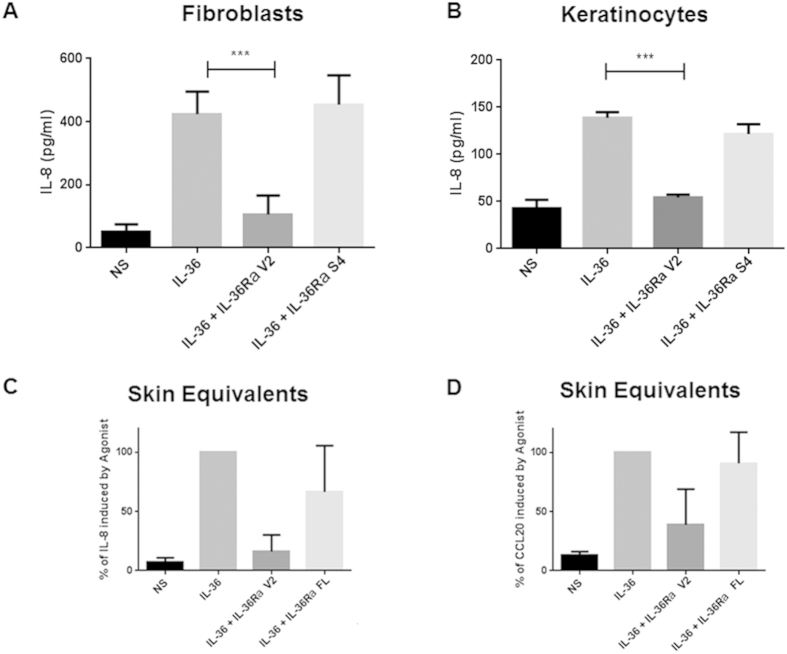
IL-36Ra requires removal of the N-terminal methionine to enable antagonistic activity. Human primary fibroblasts or keratinocytes were treated with IL-36Ra V2 and IL-36Ra S4 in the presence of IL-36 agonist (10 nM). After 48 hours of treatment IL-8 concentrations analysed by ELISA. (**A**) Fibroblasts were stimulated with IL-36Ra V2 or IL-36Ra S4 in the presence of IL-36 agonist, n = 4. (**B**) Keratinocytes were stimulated with IL-36Ra V2 or IL-36Ra S4 in the presence of active IL-36 agonist. n = 4. Mean +/− SEM is depicted on all graphs. NS = non-stimulated, FL = full length. (**C**) EpiSkin epidermal skin rafts were treated on day 11 with full length IL-36Ra or IL-36Ra V2 at 100 nM in the presence of IL-36 agonist (100 nM). After 96 hours of treatment, IL-8 and CCL20 concentrations were analysed by ELISA. n = 2.

To compare the antagonistic properties of full length IL-36Ra and the mature form generated by elastase cleavage, primary fibroblasts and keratinocytes were treated with various concentrations of full length IL-36Ra, IL-36Ra S4 and IL-36Ra V2 30 minutes prior to stimulation with IL-36 agonist. Neither full length IL-36Ra nor IL-36Ra S4 had any noticeable antagonistic activity on the agonist control. IL-36Ra V2, however, generated a significant reduction in IL-8 production in both fibroblasts (Fig. 4A) and keratinocytes (Fig. 4B) when compared to the agonist control that followed a dose dependent pattern down to a concentration of 1 nM (Supplementary Fig S1). For all experiments boiled controls were performed for IL-36Ra V2 and IL-36Ra S4 on the basis of which we ruled out any LPS contamination. None of the IL-36Ra preparations showed cytokine inducing activity when given alone.

In addition to testing the effects of full length and mature antagonists on monolayer primary cells, their biological functions were also examined in an epidermal skin model. Skin equivalents were incubated with IL-36 agonist with or without the presence of either full length or active IL-36Ra from day 10 for 96 hours. In both monolayer cultures and skin equivalents, full length IL-36Ra exhibited no antagonistic activity, whilst the IL-36Ra V2 truncation antagonised IL-36 agonist stimulation causing a significant reduction in both IL-8 and CCL20 expression (Fig. 4C). These data suggest a role for elastase in activating IL-36Ra and the consequent regulation of IL-36 mediated inflammation.

Incubation with the recombinant proteases generated two forms of IL-36Ra; the established active IL-36Ra V2 and to a lesser extent the smaller IL-36Ra S4. IL-36Ra S4 was also tested alongside IL-36Ra V2 to see if any biological function could be observed. Whilst IL-36Ra V2 acted as predicted, IL-36Ra S4 did not inhibit IL-36 agonist mediated expression of IL-8, thus exhibits no antagonistic activity (Fig. 4A,B). This suggests further cleavage of IL-36Ra from the V2 to the S4 form deactivates the antagonist.

## Discussion

Given the high expression and importance of IL-36 activity in psoriatic inflammation, understanding the mechanisms of its regulation is an important step towards a targeted therapy^5^,^11^. The IL-36 cytokines contribute to inflammatory signalling primarily in the skin, lungs and joint tissue through activation of NF-κB, and are increasingly recognised as having a protective role in anti-fungal immune responses. Like other IL-1 family members, IL-36 cytokines have been shown to require N-terminal cleavage precisely 9 amino acids upstream of a shared A-X-D motif 7. Proteases responsible for cleavage of the proteins have proved elusive due in part to the lack of shared amino acid consensus surrounding the cleavage sites. Despite this, several neutrophil proteases have recently been identified as capable of cleaving and activating IL-36 agonists^12^. Our study has shown that a neutrophil protease is also capable of activating IL-36 receptor antagonist. We have shown that neutrophil elastase cleaves the receptor antagonist member of the IL-36 cytokines, IL-36Ra, 9 amino acids upstream of its A-X-D consensus, generating IL-36Ra V2; a biologically active form of the antagonist.

With regard to neutrophil-mediated cleavage of IL-36Ra, these data bring to light some intriguing insights into the effect neutrophils may have on IL-36 mediated inflammation. Incubation with neutrophil proteases revealed that neutrophil elastase efficiently cleaved the majority of IL-36Ra to its active IL-36Ra V2 truncation whilst prolonged incubation with cathepsin G and proteinase 3 lead to a small percentage of the antagonist being cleaved to an inactive IL-36Ra S4 truncation. The ability of neutrophil elastase to activate IL-36Ra suggests infiltrating neutrophils might promote regulation of IL-36 mediated inflammation through the production of elastase. IL-36 agonists promote the maturation of human dendritic cells and their production of inflammatory mediators and enhancement of CD4 T cell effector functions and therefore have a far-reaching influence^13^. The lack of antagonistic regulation would perpetuate a positive feedback loop whereby active IL-36 agonists stimulate dendritic cells, fibroblasts and keratinocytes to express CCL20, IL-8, IL-12, IL-17C, TNFα, and more IL-36^14^,^15^,^16^,^17^, which in turn reinforce the inflammatory environment that may give rise to generation of psoriatic plaque lesions. These data suggest infiltrating neutrophils may favour cleavage of IL-36Ra to the active V2 form, placing a dampener on the self-perpetuating feedback loop to allow a more regulatory environment to exist.

In addition to their role in psoriatic inflammation the epithelial derived IL-36 cytokines have been implicated in lung pathology and shown to affect the activity of numerous immune cells. Bronchial epithelial cells strongly induce IL-36 when challenged with cigarette smoke and in response to infectious agents including Rhinovirus and *Pseudomomonas aeruginosa*^18^,^19^,^20^. Moreover mouse models have shown that administration of IL-36 leads to neutrophilic infiltrate into the bronchial alveolar fluid^21^. IL-36 agonists and IL-36Ra are also expressed in response to the respiratory fungal pathogen *Aspergillus fumigatus* suggesting a role in antifungal immunity^22^. Both IL-36γ and IL-36Ra are found to be significantly induced when incubating live *A. fumigatus* conidia and hyphae with peripheral blood mononuclear cells, and IL-36Ra expression continues to be induced by heat-killed conidia. It has also been shown that blocking the IL-36 receptor pathway by IL-36Ra in *A. fumigatus* infection causes a reduction in IL-17 and interferon-γ (IFNγ) expression; cytokines well established as important antifungal inflammatory mediators^22^. Neutrophils play a vital role in fungal immunity where a strong neutrophil response is paramount to resolution of infection. However, in addition to their phagocytic role, the neutrophils may simultaneously act in a regulatory capacity by activating local IL-36Ra, keeping IL-36 mediated inflammation in check. Furthermore, since IL-36Ra alone is induced by heat-killed conidia, neutrophils present at the latter stages of Aspergillus infection, where Aspergillus debris may induce IL-36Ra expression, may promote resolution of inflammation through elastase mediated activation of IL-36Ra.

Whilst IL-36Ra may also be activated by an intracellular mechanism analogous to IL-1β processing, there have been multiple documented examples of IL-1 family members becoming activated by extracellular neutrophil proteases after release of their pro-forms. Within the inflammatory environment, many cells will undergo pyroptosis and necrosis where cell swelling and membrane rupture results in the release of unprocessed IL-1 family members exposing them to extracellular proteases. Numerous neutrophil proteases have been shown to process and activate IL-1α, IL-1β, and IL-33. IL-1α has been shown to be processed and activated by neutrophil proteases with a comparable efficiency to that of calpain and granzyme B, and is detectable in bronchoalveolar lavage fluids from patients with cystic fibrosis^23^. Both neutrophil elastase and cathepsin G have been shown to cleave and activate IL-1β, albeit to a lesser extent than caspase-1, yet disease progression of neutrophil-dominated forms of arthritis in *Casp1*^−/−^ is comparable to that in wild type mice, suggesting a functional role for other enzymes in the regulation of IL-1β^24^,^25^,^26^. Neutrophil elastase and cathepsin G also cleave and activate extracellular IL-33 to an extent that is detectable in bronchoalveolar lavage fluid from mice with neutrophil-dominated acute lung injury^27^. Neutrophil elastase activation of IL-36Ra may therefore be of importance in an inflammatory context, where unprocessed IL-36Ra may be released via pyroptosis and activated by infiltrating neutrophils.

Understanding that neutrophils may activate IL-36Ra via neutrophil elastase gives spectrum for manipulating IL-36Ra activity and hence IL-36 mediated inflammation, at either the cellular or molecular level. This may potentially yield an area where therapeutic intervention might be possible. However, due to a lack of appropriate reagents important questions still remain unanswered. The ratio of active to inactive IL-36 and IL-36Ra in healthy and diseased tissue is unknown. It is also unclear whether extracellular availability of IL-36Ra or excessive protease cleavage are limiting factors in the resolution of inflammation. These data also raise the question as to why, if neutrophils activate IL-36Ra, there is such unchecked IL-36-mediated inflammation in psoriasis when psoriatic lesions are well characterised to have increased infiltration of neutrophils. In order to answer these important questions better IL-36 protein detecting reagents distinguishing between the active and inactive forms need to be developed.

## Materials & Methods

### Generation of IL-36 proteins

Constructs of full length IL-36Ra, IL-36Ra V2, IL-36Ra S4 and IL-36γ S18 with N-terminal SUMO tags were generated and expressed in *BL21 (DE3) E. coli.* Protein for use in cleavage assays was purified by Ni^2+^-affinity chromatography and size exclusion chromatography. Proteins used for stimulations were purified by Ni^2+^-affinity chromatography prior to cleavage of N-terminal SUMO by the Ulp1 protease, followed by further ion exchange and size exclusion chromatography.

### Cell culture and stimulations

Primary fibroblasts, keratinocytes and neutrophils were cultured in DMEM (GE Healthcare), KGM-2 (PromoCell) and RPMI (Gibco) respectively. In order to produce supernatant for cleavage assays, cells were grown in serum free media or Opti-MEM (Gibco) to avoid inhibition of any active proteases. Immediately after isolation, neutrophils were stimulated with 100 ng/ml PMA for 1 hour at 37 °C prior to removing cells by centrifugation and harvesting supernatant. For analysis of IL-36Ra’s antagonistic capabilities, fibroblasts and keratinocytes grown to 70% confluence in DMEM or KGM-2 minus EGF and hydrocortisone were stimulated with IL-36γ S18 15 minutes after addition of IL-36Ra. Supernatants were harvested 48 hours after stimulation for analysis by ELISA.

### Skin equivalents

Epidermal skin equivalents (EpiSkin) were purchased from SkinEthics and maintained according to the manufacturer’s protocol. Skin equivalents were treated on day 11 with either full length or active IL-36Ra with or without IL-36γ S18 for 96 hours. Supernatants were harvested on day 15 for analysis of IL-8 and CCL20 expression measured by ELISA.

### Cleavage assays, SDS PAGE and western blotting

1–2 μg of SUMO tagged IL-36Ra were incubated with supernatants from primary cells at 37 °C for 1 hour with or without protease inhibitors (IAA 50 μM), EDTA (500 μM), PMSF (1 mM), 1x PI Roche, α1 anti-trypsin (1 mg/ml) were used at concentrations outlined by the manufacturers. Incubations performed with recombinant proteases (proteinase 3, human neutrophil elastase, cathepsin G (Calbiochem)) were set up as recommended by manufacturers and incubated at 37 °C for up to 5 hours. Cleavage was analysed by SDS PAGE or WB using monoclonal anti-IL-36Ra antibody (R&D systems). Cleaved products were further analysed by in-house Liquid Chromatography Mass Spectrometry or analysed via N-terminal sequencing.

### Enzyme-linked Immuno-Sorbent Assay

Supernatants collected from stimulated cells were tested for concentrations of IL-8 and CCL20 by ELISA kits from BioLegend and R&D system, respectively. ELISAs were carried out according to protocols provided by the manufacturers.

### Statistical analysis

Statistical significance was determined using repeated measures one way ANOVA and Tukey’s multiple comparisons test using GraphPad Prism software (GraphPad Software Inc. La Jolla, CA, USA). All data are expressed as means ± SEM. n represents independent experiments with cells from different donors. **denotes p < 0.01; ***denotes p < 0.0001.

### Ethics

All human samples were taken in accordance with the Declaration of Helsinki and participants gave their written informed consent (REC number: 11/YH/0368). All experimental protocol, method and recruitment of healthy volunteers were carried out based on ethical approval by the University of Leeds (BIOSCI09-001). Participating volunteers gave their written informed consent.

## Additional Information

**How to cite this article**: Macleod, T. *et al*. Neutrophil Elastase-mediated proteolysis activates the anti-inflammatory cytokine IL-36 Receptor antagonist. *Sci. Rep.* **6**, 24880; doi: 10.1038/srep24880 (2016).

## Supplementary Material

Supplementary Information

## Figures and Tables

**Table 1:** Summary of cleavage products identified by mass spectrometry and N-terminal sequencing.

**IL-36RA**	**Elastase**	**Proteinase 3**	**Cathepsin G**	**PMN SN**
V2	x			
L3				x
S4		x	x	x

Depicted are all the cleavage products identified by mass spectrometry and N-terminal sequencing following incubation with recombinant serine proteases (elastase, proteinase 3 and cathepsin G) as well as with activated PMN supernatant (PMN SN). The IL-36 cleavage products are named by the new N-terminal amino acid after cleavage.
