# Supplementary material for: Neutrophil Elastase-mediated proteolysis activates the anti-inflammatory cytokine IL-36 Receptor antagonist
Source: Sci Rep. 2016 Apr 22;6:24880. doi: 10.1038/srep24880 (PMC4840362; doi:10.1038/srep24880)
Supplement: Supplementary Information [file srep24880-s1.pdf]

Neutrophil Elastase-mediated proteolysis activates the anti-inflammatory cytokine IL-36 Receptor antagonist.

Tom Macleod[1], Rosella Doble[1], Dennis McGonagle[2,3], Christopher W. Wasson[1], Adewonuola Alase[2], Martin Stacey[1], Miriam Wittmann*[2,3,4,]

[1]School of Molecular and Cellular Biology, Faculty of Biological Sciences, University of Leeds, Leeds, UK

[2]Leeds Institute of Rheumatic and Musculoskeletal Medicine (LIRMM), University of Leeds, UK

[3]National Institute of Health Research (NIHR) LMBRU, Chapel Allerton Hospital, Leeds

[4]Centre for Skin Sciences, Faculty of Life Sciences, University of Bradford, UK

*Corresponding Author:

Miriam Wittmann, MD

Chapel Allerton Hospital, LMBRU

Chapeltown Road, Leeds

LS7 4SA, UK

Email: M.Wittmann@leeds.ac.uk

Phone: ++44 113 392 4483

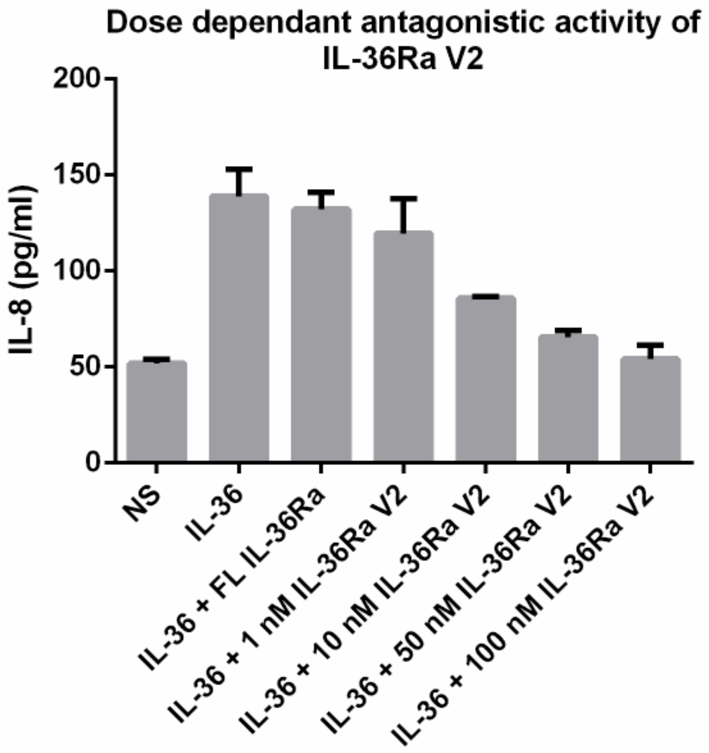

Supplementary Figure S1: Antagonistic activity of IL-36Ra V2 is dose dependant

Keratinocytes were stimulated with varying concentrations of IL-36Ra V2 in the presence of active agonist (10 nM). After 48 hours of treatment IL-8 concentrations analysed by ELISA. Mean +/- SEM is depicted. NS = non-stimulated, FL = full length. n=2.
